# ﻿Phylogenetic position, supplementary description and phytochemical analysis of *Camelliahekouensis* (Theaceae), a critically endangered tree native to Hekou, Yunnan, China

**DOI:** 10.3897/phytokeys.256.149481

**Published:** 2025-05-29

**Authors:** Dongwei Zhao, Guiliang Zhang, Shixiong Yang

**Affiliations:** 1 Department of Forestry, College of Forestry, Central South University of Forestry and Technology, Changsha, Hunan 410004, China Central South University of Forestry and Technology Changsha China; 2 Hekou Administration Branch of Dawei Mountain National Nature Reserve, Hekou, Yunnan 661399, China Hekou Administration Branch of Dawei Mountain National Nature Reserve Hekou China; 3 CAS Key Laboratory for Plant Diversity and Biogeography of East Asia, Kunming Institute of Botany, Chinese Academy of Sciences, Kunming, Yunnan 650201, China Kunming Institute of Botany, Chinese Academy of Sciences Kunming China

**Keywords:** Conservation, extremely small population, purine alkaloids, taxonomy

## Abstract

*Camellia* harbors unique diversity along Sino-Vietnamese border. Some species of them are under threat due to human activity. *Camelliahekouensis*, a native of Hekou, Yunnan, China, was once considered extinct as the previously known “last living tree” died in 2024. Fortunately, 11 in-situ and 32 ex-situ trees have been protected and propagated by the staff of Hekou Administration Branch of Dawei Mountain National Nature Reserve in Yunnan with their great unpublicized efforts. Molecular phylogenetic analysis suggests that *C.hekouensis* is nested in the main clade CI of *Camellia* and forms a clade with *C.corallina*, *C.gracilipes* and *C.pubicosta*, which are generally distributed in Vietnam. Morphological characters of the capsule and seed of *C.hekouensis* are supplementally described. The leaves of *C.hekouensis* contain 1.18 mg/g theobromine, which disagrees with the previous chemotaxonomic claim. Though the economic and ecological values are little known for *C.hekouensis*, the species should be conserved and propagated effectively and promptly to prevent extinction.

## ﻿Introduction

*Camellia* L. (Theaceae) is an economically valued genus that contains tea plants, oil camellia and camellias. The plants of *Camellia* are naturally distributed in East, Southeast and South Asia (Sealy 1958, Ming 2000). Species of the genus were counted as either 119 (Ming 2000) or 280 (Chang 1998). Since 2000, more than 100 species have been described (Zhao 2024a). As a shrub or tree bearing evergreen leaves, *Camellia* harbors great diversity in tropical China and Southeast Asia (Zhao et al. 2023), a global biodiversity hotspot attracting conservation interests (Meng and Song 2023).

*Camelliahekouensis* C.J.Wang & G.S.Fan was described based on a single specimen collected in Hekou, Yunnan, China, along the Sino-Vietnamese border (Wang and Fan 1988). A Latin diagnosis and description were provided in the protologue with a simplified interpretation of the morphological differences between *C.hekouensis* and *C.longissima* Hung T.Chang & S.Ye Liang in Chinese, while the description of fruit and seed was absent. *Camelliahekouensis* was placed in C.sect.Longissima Hung T.Chang in the protologue, probably because the species bore a relatively long pedicel. Camelliasect.Longissima proposed in Chang (1981) includes only two species, viz. *C.gracilipes* Merr. ex Sealy and *C.longissima*. Chang (1998) subsequently confirmed that C.sect.Longissima included *C.hekouensis* and stated that both caffeine and theobromine were absent from the leaves of the species in the section. Ming (1999) recognized C.sect.Longissima as a heterotypic synonym of C.sect.Longipedicellata Hung T.Chang and transferred *C.hekouensis* to the latter with *C.gracilipes*, *C.laotica* (Gagnep.) T.L.Ming, *C.longipedicellata* (Hu) Hung T.Chang & D.Fang and *C.longissima*. Successive gatherings of *C.hekouensis* have been collected from the same locality as the type specimen (Ming 2000), and the morphology of fruit and seed of the species remains unclear (Ming and Bartholomew 2007). Gao et al. (2005: 164) listed an account and a photo of *C.hekouensis* that was introduced to the International *Camellia* Species Garden at Jinhua, Zhejiang, China; the photo is, however, actually a plant of C.sect.Thea. (L.) Griff., probably C.sinensis(L.)Kuntzevar.assamica (Royle ex Hook.) Steenis, rather than *C.hekouensis*. Zhang et al. (2016: 255–256) provided ten photos of living plants of *C.hekouensis*, which included three of the fruit and seed. Unfortunately, the rulers or scales are absent in the photos, and a detailed lingual description of the fruit and seed is awaiting addition.

Fang et al. (2010) explored the phylogenetic relationship of *C.* sections *Chrysantha* Hung T.Chang, *Longipedicellata* and *Longissima* using four plastid DNA regions for *C.hekouensis* and another 27 species of *Camellia*. Their analysis suggested that *C.hekouensis* was sister to all other samples of *Camellia*, including *C.longipedicellata* and *C.longissima*, which implies that neither C.sect.Longipedicellata nor C.sect.Longissima is a monophyletic group and *C.hekouensis* occupies a unique but less resolved phylogenetic position in *Camellia*. Pang et al. (2022) suggested that *C.hekouensis* was nesting with species of C.sect.Thea based on phylogenetic analysis using four plastid DNA regions. Their samples were all collected from the International *Camellia* Species Garden at Jinhua, Zhejiang, China, and the sample of *C.hekouensis* was collected on 28 December 2006. However, the collection of *C.hekouensis* used in the analysis of Pang et al. (2022) was probably misidentified as discussed above for the species shown in Gao et al. (2005: 164), and the first author visited the garden in March 2018 but did not find any living or processed sample of *C.hekouensis* there. Thanks to the poor availability of the DNA samples, *C.hekouensis* is generally absent from previous phylogenetic analyses based on nuclear DNA (Vijayan et al. 2009, Yan et al. 2021, Cheng et al. 2022, Zan et al. 2023, Zhao et al. 2023).

**Figure 1.**
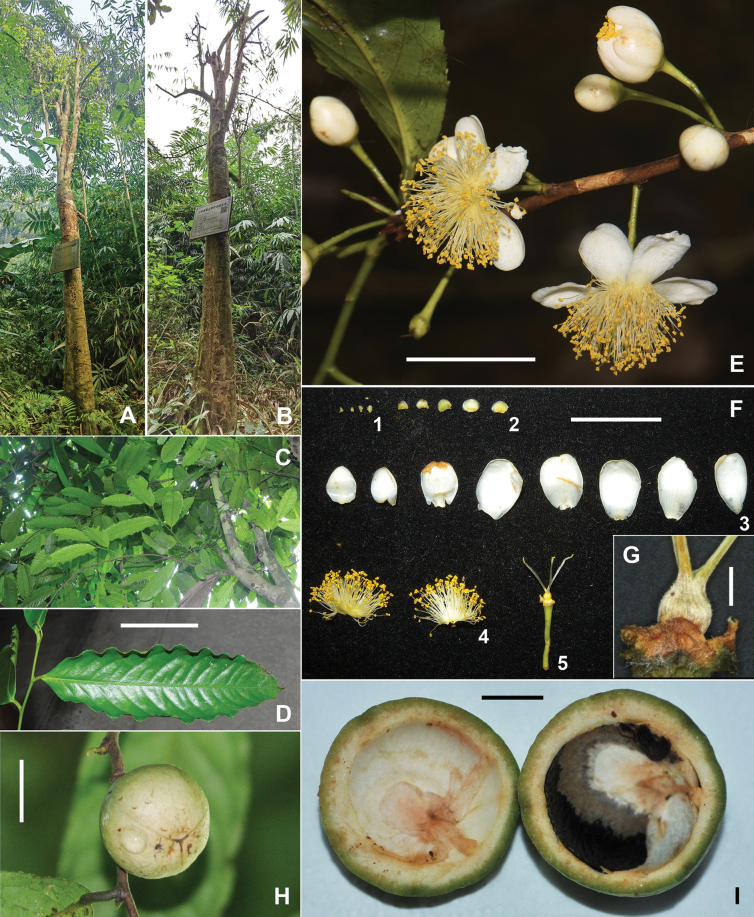
*Camelliahekouensis***A** habit of the previously misidentified “last living tree” **B** the tree was found to have died at the end of 2024 **C** branches and leaves **D** adaxial surface of a leaf **E** flowers **F** a dissected flower 1 bracteoles 2 sepals 3 petals 4 androecium 5 pedicel, receptacle and gynoecium **G** dry ovary **H** capsule **I** a dissected capsule with a single seed. Photos: S.X. Yang (**A, B, F, G**); D.W. Zhao (**C, D**); G.L. Zhang (**E, H, I**). Scale bars: 5 cm (**D**); 2 cm (**E**, **F, H**); 1 mm (**G**); 1 cm (**I**).

A tree previously known as the “last living tree” of *C.hekouensis* was found to have died at the end of 2024 due to the destruction of its bark (Fig. 1A, B). Fortunately, the staff of Hekou Administration Branch of Dawei Mountain National Nature Reserve (hereafter the Reserve) in Yunnan have made great unpublicized efforts to propagate the plants and currently maintain dozens of living plants of the species. A complete morphological description, including those of fruit and seed, is provided here for the species. The phylogenetic position of *C.hekouensis* in the genus is discussed and the chemical contents of its leaves are analyzed to uncover its potential value for horticulture before it is too late to conserve this unique species of *Camellia*.

## ﻿Material and methods

Fieldworks were undertaken to search the living individuals of *C.hekouensis* in Hekou and neighboring counties of Yunnan. Photos of the habitat and fresh characters of the vegetative and propagative organs were taken. Clean and young leaves were collected and dried in silica gel, then preserved at −30 °C for total DNA extraction and chemical analysis. Voucher specimens were deposited at the herbaria shown in Suppl. material 1. Morphological characters were described based on living plants, their images or herbarium specimens conserved in CSFI, KUN, SWFC and SYS (acronyms following Thiers 2025, continuously updated).

Total genomic DNA extraction and subsequently molecular phylogenetic analyses using three nuclear regions, including *RPB2* introns 11–15 and 23 and *waxy*, were generally stated in Zhao et al. (2023), except the GTRGAMMA model was used for bootstrapping in the maximum likelihood analysis and each metropolis-coupled Markov chain Monte Carlo analysis was run for ten million generations in the Bayesian inference. The GenBank accession numbers are listed in Suppl. material 1.

The contents of total polyphenols and catechins of dry samples, including catechin (C), epicatechin (EC), epicatechin gallate (ECG), epigallocatechin (EGC), epigallocatechin gallate (EGCG), and gallic acid (GA), were detected based on the National Standard of the People’s Republic of China, Determination of total polyphenols and catechins content in tea (GB/T 8313–2018). The contents of four purine alkaloids, including caffeine, theobromine, theophylline, and theacrine, were analyzed using high-performance liquid chromatography (HPLC) methods stated in Meng et al. (2018). Each chemical was analyzed with two replications and the mean value was calculated as the final content.

## ﻿Results

### ﻿Living trees of *C.hekouensis*

Eleven trees of *C.hekouensis* have been found in the natural forests in Hekou, Yunnan, China, including three adult trees that can bear flowers and occur dispersedly and individually, accompanied by eight juvenile trees. Meanwhile, the staff of the Reserve have successfully propagated 32 juvenile trees by seeds. Detailed localities of the living trees are unrevealed here for conservation reasons.

### ﻿Phylogenetic position

**Figure 2.**
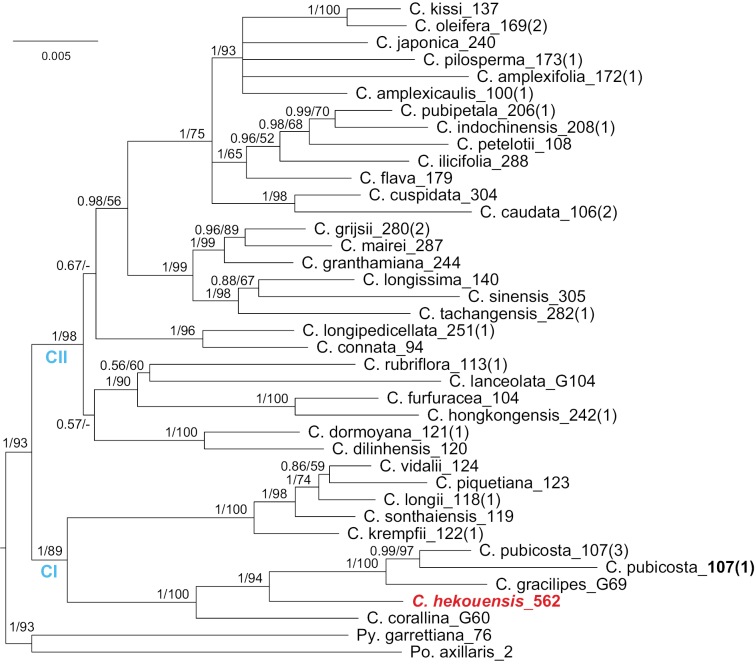
The Bayesian consensus tree reconstructed by the concatenated *RPB2* (introns 11–15 and 23) and *waxy* data for *Camelliahekouensis* and other representative species of *Camellia*. Bayesian posterior probabilities (PP) ≥ 0.5 and Bootstrap values (BS; %) ≥ 50 are presented above or below branches as PP/BS. Branch lengths are proportional to the expected nucleotide substitutions per site. Numbers in bold indicate the samples were sequenced here.

Nuclear DNA of *RPB2* (introns 11–15 and 23) and *waxy* regions of a single sample of *C.hekouensis* and one of *C.pubicosta* Merr. were sequenced in this study (Suppl. material 1). These, and another 37 samples reported in Zhao et al. (2023), were employed to reconstruct a phylogenetic tree of *Camellia* provided in Fig. 2. It suggests that *C.hekouensis* is nested in a monophyletic group with *C.corallina* (Gagnep.) Sealy, *C.gracilipes* and *C.pubicosta* with strong support (Bayesian posterior probability [PP] = 1, Bootstrap [BS, %] = 100). They are nested in the main clade CI of *Camellia*. *Camelliahekouensis* is sister to *C.gracilipes* and *C.pubicosta* (PP = 1, BS = 94), which suggests a closely phylogenetic relationship between them. Both *C.longissima*, the type of C.sect.Longissima, and *C.longipedicellata*, the type of C.sect.Longipedicellata, are nested in the main clade CII of *Camellia*, which have a relatively remote relationship with *C.hekouensis* (Fig. 2).

### ﻿Chemical contents

The contents of catechins (C, EC, ECG, EGC, EGCG), gallic acid (GA), total polyphenols and four purine alkaloids of *C.hekouensis* are listed in Table 1. *Camelliahekouensis* has 5.13 mg/g catechins and 124.78 mg/g total polyphenols. Caffeine has not been detected, and theobromine occupies 1.18 mg/g in the dry leaves of *C.hekouensis*.

**Table 1.** Chemical contents of the leaves of *Camelliahekouensis*.

Chemical compounds	content (mg/g, ± standard deviation)
Total polyphenols	124.78 ± 3.13
Catechin (C)	2.24 ± 0.39
Epicatechin (EC)	1.84 ± 0.26
Epicatechin gallate (ECG)	0.29 ± 0.00
Epigallocatechin (EGC)	0.64 ± 0.05
Epigallocatechin gallate (EGCG)	0.12 ± 0.01
Total catechins	5.13 ± 0.69
Gallic acid (GA)	0.45 ± 0.02
Caffeine	ND
Theacrine	0.28 ± 0.03
Theobromine	1.18 ± 0.05
Theophylline	0.05 ± 0.00

Note. ND: not detected.

### ﻿Description

#### ﻿ Camellia hekouensis

Taxon classificationPlantaeEricalesTheaceae

C. J.Wang & G.S.Fan, Acta Bot. Yunnan. 10(3): 365. 1988.

A6AACA57-E169-5535-9A9E-863D5D9D5AFD

##### Type material.

**Figure 3.**
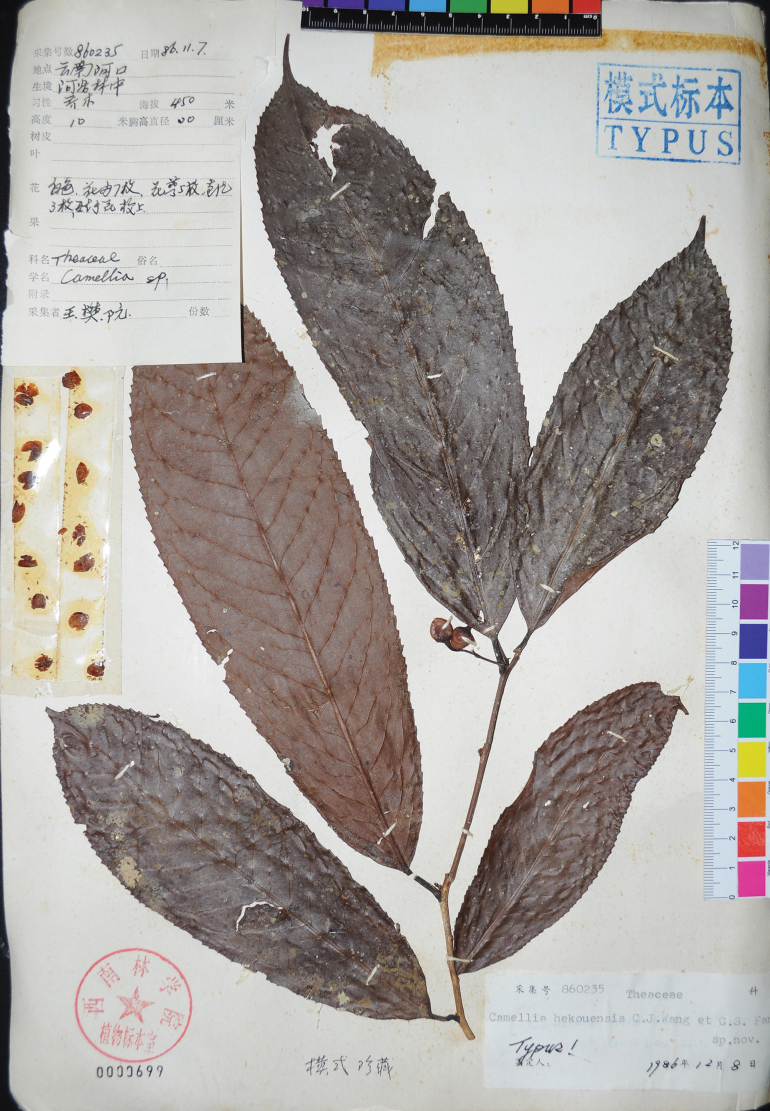
Holotype of *Camelliahekouensis* at SWFC. Photo: D.W. Zhao.

***Holotype***: China • Yunnan: Hekou, 450 m, 7 November 1986, *C.J. Wang et al. 860235* (SWFC!, Fig. 3; isotype: SWFC!).

##### Description.

Trees or shrubs up to 7 m tall, evergreen. ***Trunk*** brownish grey, ***new branchlets*** and ***terminal buds*** glabrous. ***Petioles*** 5–15 mm long, glabrous; ***leaf blades*** elliptic, obovate to oblong, 10–22 × 3–8 cm, thinly coriaceous, abaxially yellowish green, adaxially dark green, shiny, glabrous on both surfaces, midrib and secondary veins abaxially elevated and adaxially impressed, secondary veins 10–17 on each side of midrib, base cuneate to obtuse, margin serrulate, apex acuminate to caudate. ***Flowers*** axillary, fragrant, solitary or up to 3 in a cluster, ca. 3 cm in diam. ***Pedicels*** 15–25 mm long. ***Bracteoles*** 3–4, alternate, persistent, deltate to ovate, 1–1.5 × 1.5–2 mm, glabrous on both surfaces. ***Sepals*** 5, deltate to sub-orbicular, 2.5–4 × 2–3 mm, glabrous on both surfaces, margin membranous and ciliolate. ***Petals*** 7–9 in 2 whorls, white, ovate, elliptic or obovate, 6–12 × 9–15 mm, glabrous on both surfaces, basally connate for 1–2 mm. ***Stamens*** 5–11 mm long; filaments light yellow, glabrous, basally connate for ca. 1 mm and outer filaments basally adnate to petals for 1–2 mm. ***Ovary*** ovoid, densely pubescent. ***Styles*** 3, distinct, 6–9 mm long, basally sparsely pubescent and gradually becoming glabrous apically. ***Capsule*** globose, 3–4 cm in diam., 1-loculed with 1 seed; pericarp 2–6 mm thick. ***Seeds*** fuscous, globose, 2–2.5 cm in diam., glabrous. Fig. 1.

##### Phenology.

Flowering December, fruiting August.

##### Distribution and habitat.

*Camelliahekouensis* is native to Hekou, Yunnan, China and occurs in the tropical evergreen forests at elevations of 290–800 m.

##### Additional specimens examined.

Yunnan: • Hekou, Nanxi, 360–410 m, 21 December 1986, *C.J. Wang & L.S. Xie 904* (KUN 694698); • Hekou, Nanxi, 22.68°N, 103.93°E, 297 m, 27 November 2023, *D.W. Zhao et al. 562* (CSFI, equals to *S.X. Yang et al. 7352* at KUN); • same locality, 26 December 2023, *S.X. Yang et al. 7360* (KUN).

## ﻿Discussion

### ﻿Taxonomic significance

A relatively long pedicel is valued in the taxonomies of Chang (1981, 1998) and Ming (2000, Ming and Bartholomew 2007). Zhao et al. (2023) suggested that *C.longissima* formed a clade with taxa of C.sect.Thea and subsequently, Zhao (2024b) treated the former as a new member of the latter. Accordingly, C.sect.Longissima became a heterotypic synonym of C.sect.Thea. Neither Chang’s (1981, 1998) nor Ming’s (2000, Ming and Bartholomew 2007) C.sect.Longipedicellata was supported by molecular phylogenetic analyses (Fang et al. 2010, Zan et al. 2023, Zhao et al. 2023). Our phylogenetic analysis suggests that *C.hekouensis* is nested in the main clade CI of *Camellia* (Fig. 2), which mainly consists of species native to Vietnam and splits with other camellias in the early evolutionary history of the genus (Zhao et al. 2023). *Camelliahekouensis* may be placed in the clade Corallina suggested in Zhao et al. (2023) provisionally. The clade contains *C.corallina*, *C.gracilipes*, *C.hekouensis* and *C.pubicosta* as shown in Fig. 2. The latter three species are morphologically closely related and can be easily distinguished from *C.corallina* by the morphology of leaves and flowers (Ming 2000). We propose a comprehensive taxonomic revision to evaluate whether the clade Corallina should be considered to be a section in *Camellia* as a whole or may be divided into two different sections.

Chang (1998) suggested that both caffeine and theobromine were absent from *C.hekouensis* and *C.longissima*. The single sample of *C.hekouensis* is caffeine-free, but contains 1.18 mg/g theobromine (Table 1), which disagrees with the claim of Chang (1998). Chang valued the variation of chemical contents, especially purine alkaloids, in the taxonomy of *Camellia* as insisting that *C.irrawadiensis* Barua was a distinct species from *C.taliensis* (W.W.Sm.) Melch. because the former was caffeine-free (Chang et al. 1996). By contrast, Ming (2000) and Zhao (2024a, 2024b) treated the former as a heterotypic synonym of the latter. Chemical contents may be nutrition-dependent and usually vary between individuals and populations within a single species of *Camellia* (Ye et al. 1997, Zhao 2024a). They are useful to act as essential traits to select the characteristic germplasms of tea plants; great caution is, however, needed when using them overwhelmingly as the taxonomically diagnostic characters in *Camellia*.

The image of a specimen on the website of Chinese Virtual Herbarium, *T.L. Ming et al. 224* (SYS 00089904, https://www.cvh.ac.cn/spms/detail.php?id=e87563d0), was identified as *C.hekouensis* by both T.L.Ming (on 10 May 1990) and Hung T.Chang (without date on the sheet). It may be, however, a plant of *Miliusa* sp. (Annonaceae). Remarkably, Ming (2000) did not cite this collection under his description of *C.hekouensis*.

### ﻿Utilization and conservation

No records of specific utilization of *C.hekouensis* have been retrieved. Local people, except those who want to conserve the species, pay little attention to the plant. Though the species contains catechins and total polyphenols (Table 1), it does not suggest that the leaves can be used as a beverage source because *C.hekouensis* has a remotely phylogenetic relationship with C.sect.Thea (Fig. 2), the group of tea plants. The seeds of all species in *Camellia* can produce oil but their qualities vary widely (Wang et al. 2007, Su et al. 2014); unfortunately, the remaining three adult trees of *C.hekouensis* bear few seeds and the top priority is propagation rather than chemical analysis using the precious seeds. Local staff of the Reserve said the flowers of *C.hekouensis* are very fragrant, and these may be useful in horticulture. However, its white petals (Fig. 1E, F) might attract little attention of *Camellia* enthusiasts, who usually prefer yellow and red camellias. The woods of *Camellia* are usually dense and can be used for handcrafts (Mou et al. 2007). The specific wood quality of *C.hekouensis* has not been studied probably because of unavailability. Its ecological values, such as co-evolution with its pollinators, herbivores and dwellers, are barely known.

However, *C.hekouensis* represents a unique germplasm of *Camellia* (Fig. 2). The tree seems to have a relatively long life cycle. Though the adult trees can bear many flowers, the fruits are very few. Artificial pollination may be helpful to increase the seed yield. Asexual reproduction by cuttings turned out a very low rate of survival in previous trials. Nevertheless, conservation and propagation should bear the top priority for *C.hekouensis*. The death of the previously misidentified “last living tree” of *C.hekouensis* (Fig. 1B) did give us cause for alarm and brought home the fragility of the species.

## Supplementary Material

XML Treatment for Camellia hekouensis

## ﻿Additional information

### Author ORCIDs

Dongwei Zhao https://orcid.org/0000-0002-7761-7127

Shixiong Yang https://orcid.org/0000-0001-8472-1275

### Data availability

All of the data that support the findings of this study are available in the main text and GenBank as shown in Suppl. material 1.

## Supplementary materials

Supplementary material 1 Samples used in the study Data type xlsx Explanation note Vouchers and GenBank numbers of samples used https://binary.pensoft.net/file/1341256This dataset is made available under the Open Database License (http://opendatacommons.org/licenses/odbl/1.0/). The Open Database License (ODbL) is a license agreement intended to allow users to freely share, modify, and use this Dataset while maintaining this same freedom for others, provided that the original source and author(s) are credited.Dongwei Zhao, Guiliang Zhang, Shixiong Yang
